# Draft Genome Sequences of Five Staphylococcus aureus Strains Isolated from Clinically Healthy Cows in the Russian Federation

**DOI:** 10.1128/genomeA.00275-18

**Published:** 2018-04-19

**Authors:** Ksenia K. Fursova, Olga A. Artem’eva, Daria A. Nikanova, Andrey K. Larin, Natalia A. Zinovieva, Fedor A. Brovko

**Affiliations:** a L. K. Ernst Federal Science Center for Animal Husbandry, Federal Agency of Scientific Organizations, Dubrovitsy, Russian Federation; b Shemyakin and Ovchinnikov Institute of Bioorganic Chemistry, Federal Agency of Scientific Organizations, Pushchino, Russian Federation; c Federal Research and Clinical Center of Physical-Chemical Medicine, Federal Medical Biological Agency, Moscow, Russian Federation

## Abstract

We present here the draft genome sequences of five Staphylococcus aureus strains isolated from milk samples from clinically healthy cows in the Russian Federation. Four of them were determined to be sequence type 97 (ST-97), and one was determined to be ST-22. All the strains are characterized by their genome possessing genes that code for enterotoxins and cytotoxins.

## GENOME ANNOUNCEMENT

Staphylococcus aureus is a component of normal human and animal microflora. It is now often associated as a cause of mastitis development (1, 2). Mastitis is a common infectious disease of dairy cattle. Factors such as environmental temperature, microbial association, and a cow’s lactation stage can trigger a switch of the bacterium into a pathogenic form (3, 4).

Here, we report the draft genome sequences of S. aureus isolated from the milk of clinically healthy Holstein cows. Milk samples were collected from farms in different regions of the Russian Federation. Five strains were selected for whole-genome sequencing. DNA samples were extracted using the lysostaphin and GenElute bacterial genomic DNA kit (Sigma-Aldrich, Merck, Germany). Whole-genome sequencing was performed using the Ion Torrent semiconductor technique with an Ion Proton sequencer, with coverage depths from 114 to 186× for each genome. *De novo* contig assembly was performed using Newbler GS *de novo* assembler v.3.0. As a result, we obtained 51 to 56 contigs for each genome (Table 1). The genome sizes ranged from 2.69 to 2.73 Mb. Each genome contains 2,796 to 2,855 genes. The assembled contigs were annotated using the Rapid Annotation using Subsystem Technology (RAST) v.2.0 server (http://rast.nmpdr.org) (5) and the NCBI Prokaryotic Genome Annotation Pipeline (PGAP; https://www.ncbi.nlm.nih.gov/genomes/static/Pipeline.html). In genotyping analysis, S. aureus strain 17 was classified as sequence type 22 (ST-22), and another four strains were classified as ST-97 by multilocus sequence type (MLST) analysis (https://cge.cbs.dtu.dk/services/MLST/) (6). The occurrence of prophages was analyzed by PHAST (7). All these isolates have prophages (Table 1). In addition, genes that code for the enterotoxin family and the genes that code for cytotoxins (leukocidins and hemolysins) were identified for the strains by RAST.

**TABLE 1:** Strain-identifying information and basic statistics for draft genome sequences

S. aureus strain	GenBank accession no.	Total size (bp)	No. of contigs >1,000 bp	No. of genes		MLST	No. of prophages
				Total	Coding		
17	PKMS00000000	2,709,990	59	2,815	2,013	ST-22	3 (2 regions incomplete, 1 region questionable)
42	PIVF00000000	2,691,752	54	2,796	2,268	ST-97	2 (2 regions incomplete)
241	PENB00000000	2,732,925	56	2,855	2,267	ST-97	3 (2 regions incomplete, 1 region questionable)
1113	PIOE00000000	2,719,190	51	2,837	2,169	ST-97	3 (3 regions incomplete)
11131	PIOF00000000	2,714,821	53	2,833	1,971	ST-97	3 (2 regions incomplete, 1 region intact)

Thus, despite the fact that our strains were isolated from clinically healthy cows, we identified genes for virulence factors of S. aureus associated with mastitis. A more detailed report from a full comparative genomic analysis will be included in a future publication.

### Accession number(s).

The GenBank accession numbers for these five genome sequences are listed in Table 1.
